# Sacubitril/valsartan inhibits the proliferation of vascular smooth muscle cells through notch signaling and ERK1/2 pathway

**DOI:** 10.1186/s12872-024-03764-8

**Published:** 2024-02-14

**Authors:** Congfeng Xu, Ning Zhang, Hong Yuan, Liren Wang, Yonghong Li

**Affiliations:** Department of Cardiology, The Affiliated Hospital of Qingdao University, Road No. 59 Haier, Qingdao, 266000 China

**Keywords:** Restenosis, Vascular smooth muscle cells, Sacubitril/valsartan, Notch1, Jagged1, ERK1/2

## Abstract

**Aims:**

To explore the role and mechanism of Notch signaling and ERK1/2 pathway in the inhibitory effect of sacubitril/valsartan on the proliferation of vascular smooth muscle cells (VSMCs).

**Main methods:**

Human aortic vascular smooth muscle cells (HA-VSMCs) were cultured in vitro. The proliferating VSMCs were divided into three groups as control group, Ang II group and Ang II + sacubitril/valsartan group. Cell proliferation and migration were detected by CCK8 and scratch test respectively. The mRNA and protein expression of PCNA, MMP-9, Notch1 and Jagged-1 were detected by qRT-PCR and Western blot respectively. The p-ERK1/2 expression was detected by Western blot.

**Key findings:**

Compared with the control group, proliferation and migration of VSMCs and the expression of PCNA, MMP-9, Notch1, Jagged-1 and p-ERK1/2 was increased in Ang II group. Sacubitril/valsartan significantly reduced the proliferation and migration. Additionally, pretreatment with sacubitril/valsartan reduced the PCNA, MMP-9, Notch1, Jagged-1 and p-ERK1/2 expression.

**Supplementary Information:**

The online version contains supplementary material available at 10.1186/s12872-024-03764-8.

## Significance

Sacubitril/valsartan inhibits the proliferation and migration of VSMCs induced by Ang II, which may be through Notch1/Jagged1 and ERK1/2 pathways.

**Supplementary Information:**

The online version contains supplementary material available at 10.1186/s12872-024-03764-8.

## Introduction

Ischemic heart disease is a threat to human health [1]. The introduction of percutaneous coronary intervention (PCI) has completely changed the treatment of patients with coronary heart disease. coronary stents, especially drug-eluting stents, have greatly improved the prognosis of patients. However, the complications of in-stent restenosis (ISR) seriously affect the efficacy of PCI [2].

The pathophysiological mechanism of ISR is complex, which involves vascular endothelial injury, thrombosis, inflammatory response, extracellular matrix remodeling, proliferation and migration of vascular smooth muscle cells (VSMCs), among which the proliferation and migration of VSMCs are the main pathological basis [3]. Under normal circumstances, VSMCs exist in the media of blood vessels, constitute the tissue structure and maintain vascular tension. After being stimulated by pathological factors, VSMCs change from a contractile phenotype to a synthetic phenotype and the ability of VSMCs proliferation and migration increases, which leads to thickening of the intima and the occurrence of ISR [4].

The Notch signaling pathway is mainly composed of receptors(Notch1-4), ligands (Jagged-1,2, Delta-like 1, 3, 4), CSL binding proteins, regulators and other effectors [5]. Notch receptors and ligand genes are first transcribed and translated into proteins, Notch in neighboring cells transactivate receptors release the receptor intracellular domain (NICD) [6]. Then, it shuttles to the nucleus and binds to recombination signal sequence-binding protein Jκ (RBP- Jκ) in the promoter to regulate downstream gene expression [7]. NICD can not only directly interact with Ras, but also promote ERK phosphorylation, but the mechanism has not been studied clearly. In addition, Notch signaling can also regulate the expression of related genes through the non-RBP-Jκ pathway [8]. It is an important signaling system that controls cell fate such as cell proliferation, migration and apoptosis. After vascular injury, VSMCs in the neointima express a variety of Notch signaling molecules [9].

Extracellular signal-regulated kinase (ERK), divided into ERK1 and ERK2, is one of the family members of mitogen-activated protein kinases (MAPKs) and involved in many biological reactions such as cell proliferation and differentiation, cell morphology maintenance, cytoskeleton construction, apoptosis and cell malignant transformation [10]. The basic composition of the ERK pathway is a typical three-layer cascade reaction, including Raf, MEK, and ERK. These three kinases can be activated in turn to form the Ras-Raf-MEK-ERK pathway, where Ras is an upstream activator protein [11]. ERK1/2 is activated by phosphorylation and enters the nucleus to act on transcription factors such as E1k-1, c-myc, c-fos, c-jun, ATF, NF-kB and AP-1 to promote the transcription and expression of certain genes. A number of studies have shown that the effect mediated by ERK plays an important role in the proliferation of VSMCs [12].

**Fig. 1:**
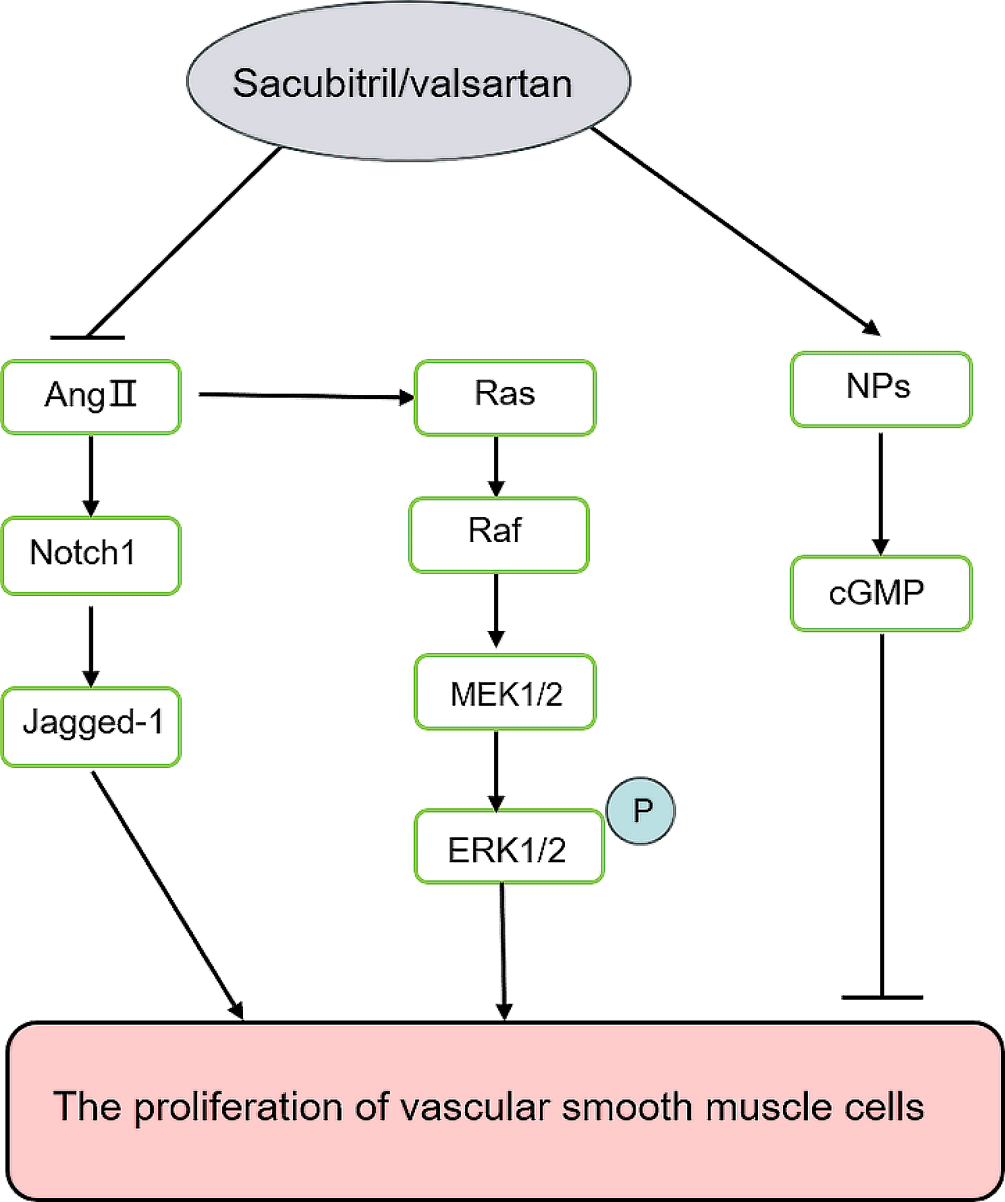
The signaling pathway of sacubitril/valsartan inhibiting VSMCs proliferation. Sacubitril/valsartan can inhibit the migration and proliferation of VSMCs induced by Ang II, which may be through Notch1/Jagged-1 and ERK1/2 pathways. On the one hand, Ang II increases the expression of Notch1 and Jagged-1, causing the proliferation and migration of VSMCs. On the other hand, it activates the ERK1/2 pathway, in which Ras is an upstream activator protein, Raf and MEK1/2 are activated in turn, resulting in increased phosphorylation of ERK1/2. In addition, sacubitril/valsartan also inhibits the decomposition of NPs, thereby inhibiting the proliferation and migration of VSMCs

Valsartan/sacubitril (Also known as the Entresto) is a combination of angiotensin II receptor/neprilysin inhibitor (ARNI) [13]. Natriuretic peptides (NPs) mainly include A-type natriuretic peptide (ANP), B-type natriuretic peptide (BNP), and C-type natriuretic peptide (CNP). After binding to the receptor, NPs increase the second messenger cGMP and act on the downstream signaling pathway, playing a role in natriuretic, inhibition of the renin–angiotensin–aldosterone system (RAAS) and anti-proliferative systems [14]. Studies have found that circulating RAAS in obese patients is dysregulated and is activated at both local and systemic levels and reduced CNPS effects coupled with increased RAAS activity have a central role in obesity. Thus, RAAS and NPs are endogenous antagonistic systems of sodium balance and cardiovascular system [15]. Neprilysin is an endopeptidase that cleaves natriuretic peptide to inactivate it, and its inhibitor ARNI can inhibit this situation [16]. In addition, valsartan can inhibit Ang II-induced proliferation of VSMCs through Mfn2-Ras-Raf-ERK/MAPK signaling pathway in vitro [17]. Studies have confirmed that valsartan/sacubitril inhibits the proliferation of human pulmonary artery VSMCs in patients with idiopathic pulmonary hypertension as well [18], but the effect on VSMCs after vascular endothelial injury has not been reported. In this study, we observed the effect of sacubitril/valsartan on the proliferation and migration of HA-VSMCs stimulated by Ang II, and studied the Notch signaling and ERK1/2 pathway (Fig. 1).

## Materials and methods

### Cell culture

Human aortic (HA)-VSMCs was obtained from iCell Bioscience (Shanghai, China) and maintained in Dulbecco’s modified Eagle’s medium (DMEM; Gibco, Grand Island, NY, USA) including 10% fetal bovine serum (Meilunbio, Dalian, China) and 1% penicillin/streptomycin (Solarbio, Beijing, China) at an atmosphere of 5% CO_2_ and 37 °C.

### Experimental groups

Ang II, sacubitril/valsartan were purchased from AbMole (Shanghai, China). Firstly, HA-VSMCs were seeded in serum-free medium in 6-well culture plates and cultured for 24 h after starvation. Secondly, the cells were divided into the following groups: Ang II group: Ang II(1 × 10^− 6^mol/L) was added directly. Ang II + Entresto group: Ang II(1 × 10^− 6^mol/L) was added after Entresto (1 × 10^− 5^mol/L) worked for 1 h. Control group: this group was added without Ang II and Entresto. Each group was treated for 24 h.

### Cell proliferation was detected by CCK8

HA-VSMCs were seeded in 96-well plates, and when the cells were fused to 80%, they were starved in serum-free medium for 24 h. Then, each group was treated with drugs and cultured for 24 h. CCK-8 reagent (Solarbio, Beijing, China) was added to each well for 2 h, and the content of it was 10%. The optical density (OD) value at 450 nm of each well was measured on a microplate reader (Thermo Fisher Scientific). Each group had 5 holes, and the experiment was repeated 3 times.

### The scratch wound assay

HA-VSMCs were inoculated into 6-well plates. When the cells grew to 70-80%, they were treated with serum-free medium for 24 h. A sterile gun head of 1000µL was used to make a uniform line on the cell plate vertically, resulting in several ‘wound’. The cells were washed with PBS three times to remove the scratched cells, and then added with 1% FBS. According to the groups, Entresto(1 × 10^− 5^mol/L) was added to incubate with the cells for 1 h, and then Ang II(1 × 10^− 6^mol/L) was added to continue the action. The migration was observed under the microscope every 12 h and the images were collected.

### Quantitative real-time reverse transcription PCR (qRT-PCR)

(1) Each group was grouped according to the above method for 24 hours, and total RNA was extracted with trizol (Invitrogen), chloroform and isopropanol. Then, the concentration and purity of RNA extracts were detected using micro spectrophotometer instrument (Thermo Fisher Scientific, USA) and the ratio of all samples was greater than 1.7. Reverse transcription was performed according to the reverse transcription kit (Agbio, Hunan, China) operation steps to obtain cDNA. The reaction conditions were as follows: a water bath at 37℃ for 15 min, 85℃ for 5 s, and finally set to 4 ℃. (2) qRT-PCR was executed with SYBR Green Premix Pro Taq HS qPCR Kit (Agbio, Hunan, China) on a LightCycler96 (Roche, Germany). The thermocycling condition was: 95℃ for 30 s; followed by 40 cycles of denaturation at 95℃ for 5 s and annealing at 60℃ for 30 s. The relative expression of PCNA、MMP-9、Notch1and Jagged-1was normalized to the level of GAPDH using the 2^−ΔΔCT^ method. PCNA forward primer was 5’-GCTAGTATTTGAAGCACCAAACCA-3’ and reverse was 5’-TCTCTCCGGCATATACGTGCAAATT-3’, MMP-9 forward was 5’ -CCCGGACCAAGGATACAGTTT-3’ and reverse was 5’ -GTGCCGGATGCCATTCAC-3’, Notch1 forward was 5’ -CGCCGTGAACAATGTGGAT-3’ and reverse was 5’ -GTCCCGGTTGGCAAAGTG-3’, Jagged-1 forward was 5’ -GGGAACCCGATCAAGGAAAT-3’ and reverse was 5’ -GCTCAGCAAGGGAACAAGGA-3’. GAPDH forward was 5’ - AAGGTCGGAGTCAACGGATT − 3’ and reverse was 5’ - AGTGATGGCATGGACTGTGG − 3’.

### Western blot

(1) After the cells in each group were washed with PBS for 3 times, RIPA lysate (Meilunbio, Dalian, China) was used to isolate total protein from HA-VSMCs, and extracted proteins were quantified by a BCA Protein Quantification Kit (Epizyme, Shanghai, China). (2) SDS-PAGE gel (Epizyme, Shanghai, China) electrophoresis was performed, and then the proteins were transferred onto polyvinylidene fluoride (PVDF) membranes (Millipore, USA) and blocked with 5% slim milk for 1 h. (3) The membranes were incubated overnight at 4 °C together with relevant primary antibodies and then incubated with corresponding secondary antibody for 2 h at room temperature. (4) The protein bands were visualized by enhanced chemiluminescence (Vazyme, Nanjing, China) and quantified by Image Lab (LI-COR). β-actin, PCNA and MMP-9 antibodies were purchased from Absin (Beijing, China), while Notch1, Jagged-1, ERK1/2 and p-ERK1/2 were from Abmart (Shanghai, China).

### Statistical analysis

All experiments were carried out three times and all values were shown as means ± SEM. Statistical analysis was performed using one-way analysis of variance (ANOVA) followed by post hoc tests (Turkey’s test). P-values of less than 0.05 presented the difference was statistically significant.

## Results

### The effects of Entresto on the Proliferation and Migration of VSMCs

#### CCK8

**Fig. 2:**
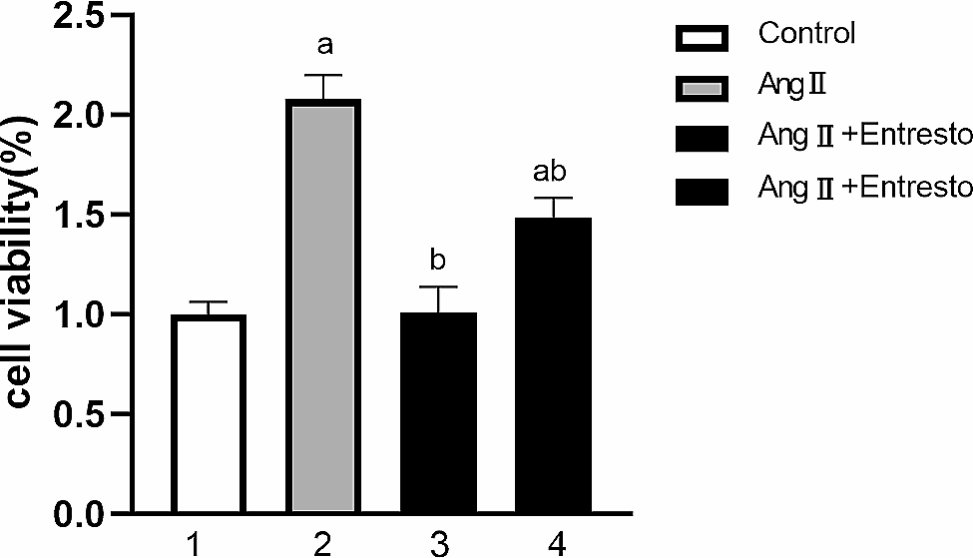
The effect of Entresto on VSMCs proliferation stimulated by AngII. 1: Control group, 2: AngII (10^− 6^mol/L) group, 3: AngII (10^− 6^mol/L) + Entresto (10^− 5^mol/L) group, 4: AngII (10^− 6^mol/L) + Entresto (10^− 6^mol/L) group. (1) Entresto was used for 1 h before Ang II stimulation for 24 h, and CCK8 was used to detect the cell inhibition rate of each group. a: *p* < 0.05, compared with the control group. b: *p* < 0.05, compared with Ang II group

Our data shown in Fig. 2 revealed that the OD of the control group, Ang II group, Ang II + Entresto(1 × 10^− 5^mol/L) group and Ang II + Entresto(1 × 10^− 6^mol/L) group were 1.00 ± 0.02, 2.08 ± 0.05, 1.01 ± 0.05 and 1.49 ± 0.04 respectively. There was significant difference among the groups (F = 115.1, *P* < 0.01). Compared with the control group, Ang II could significantly stimulate the proliferation of VSMCs (t = 17.67, *P* < 0.01). Compared with Ang II group, Entresto at the concentration of 10^− 5^~10^− 6^ mol/L significantly inhibited the proliferation of VSMCs promoted by Ang II (t = 8.45,13.55, *P* < 0.05).

#### The scratch wound assay

**Fig. 3:**
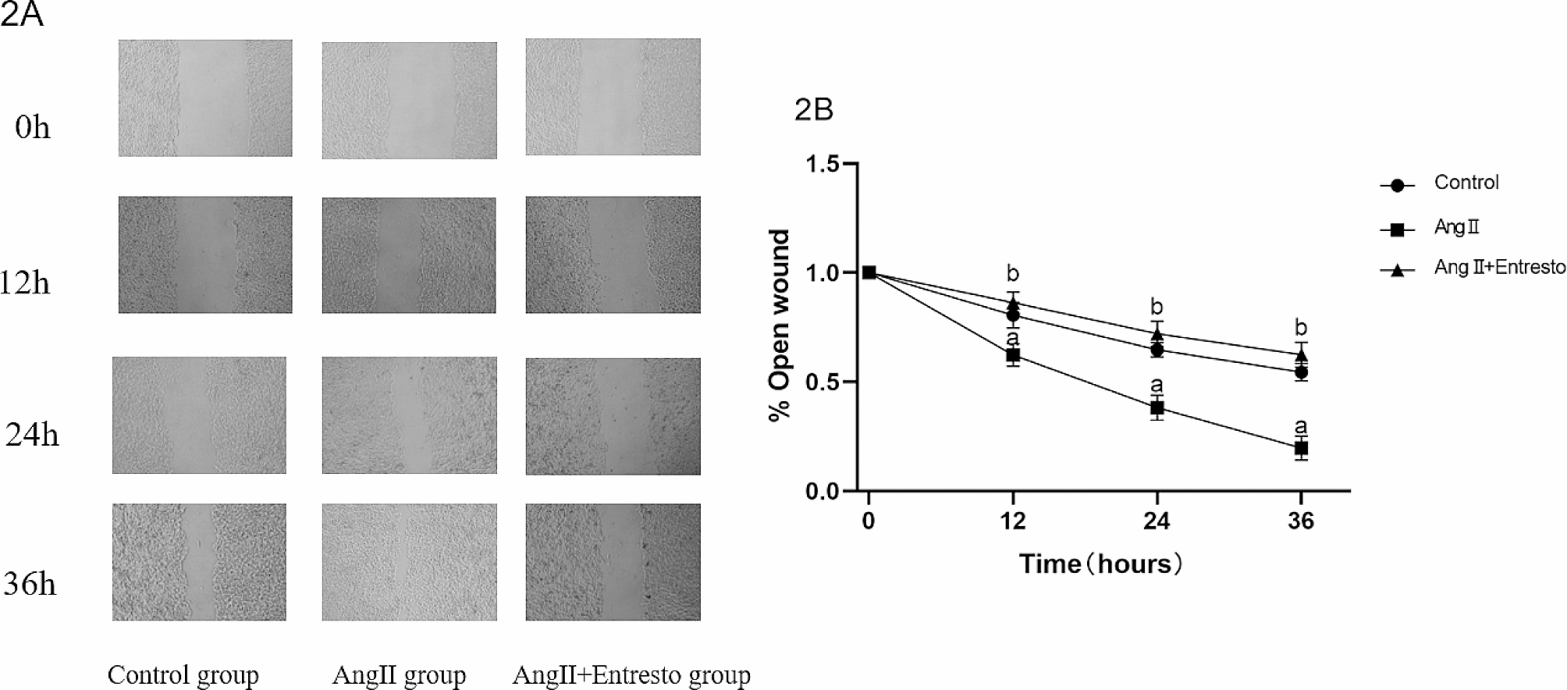
The effect of Entresto on VSMCs migration stimulated by AnII. 1: Control group, 2: AngII (10^− 6^mol/L) group, 3: AngII (10^− 6^mol/L) + Entresto (10^− 5^mol/L) group. (**2A**) Entresto was applied for 1 h before Ang II stimulation for 24 h, and the migration distance of VSMCs was detected. (**2B**) This is the quantization diagram. a: *p* < 0.05, compared with the control group, b: *p* < 0.05, compared with Ang II group

The scratch wound assay showed that there was no significant difference in the degree of cell migration between the groups at 0 h (*P* > 0.05), and the difference in cell migration ability between the groups at 12 h ~ 36 h was statistically significant (F = 16.54 ~ 58.64, *P* < 0.01). Compared with the control group, the migration activity of VSMCs in Ang II group was significantly increased at 12 h ~ 36 h (t = 4.04 ~ 8.78, *P* < 0.05). Compared with Ang II group, the migration activity of VSMCs in Ang II + Entresto group was significantly decreased at 12 h ~ 36 h (t = 5.79 ~ 9.32, *P* < 0.01) (Refer to Fig. 3).

#### The gene and protein expression of PCNA and MMP-9 in VSMCs

**Fig. 4:**
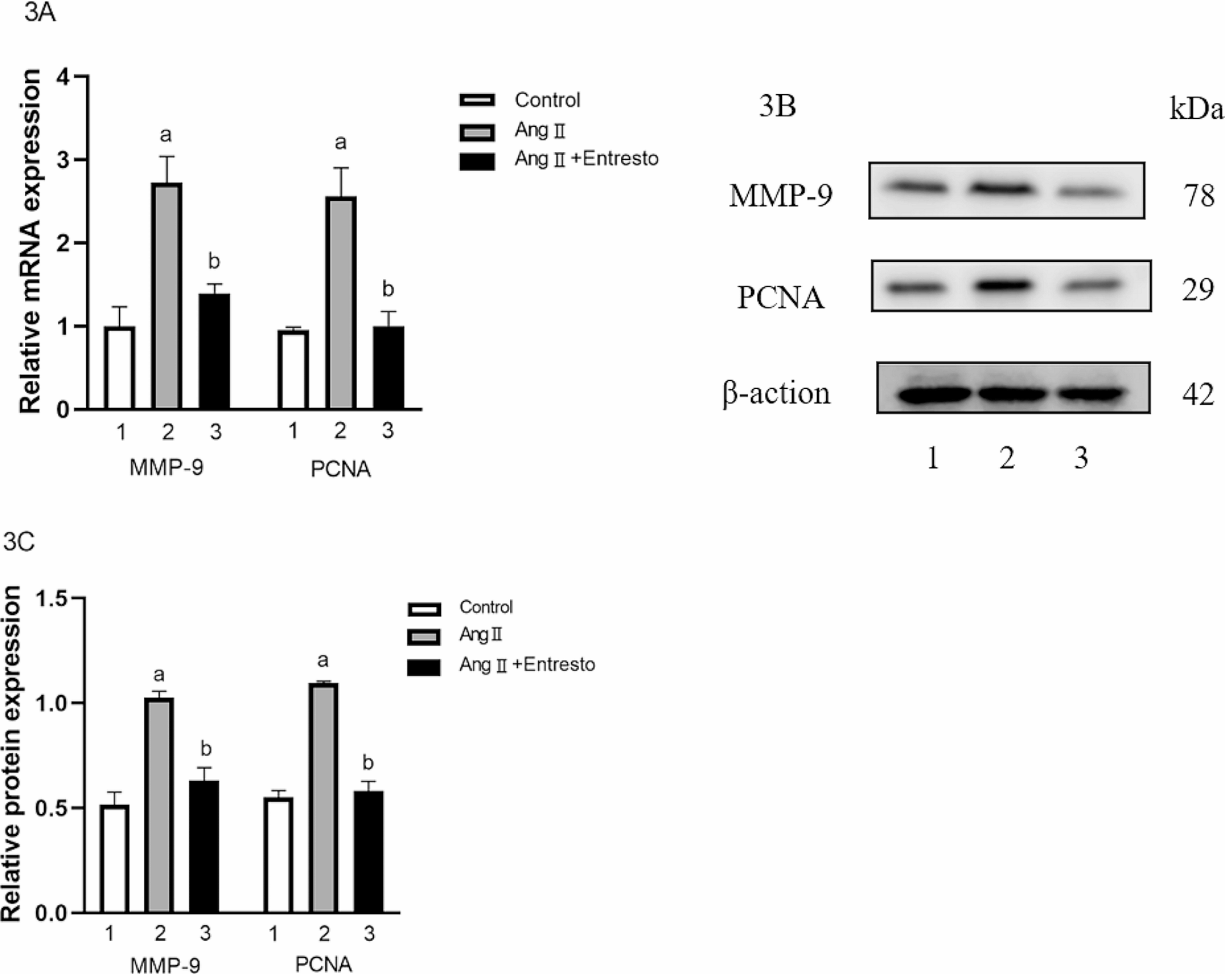
The expression of PCNA and MMP-9 in VSMCs. 1: Control group, 2: AngII (10^− 6^mol/L) group, 3: AngII (10^− 6^mol/L) + Entresto (10^− 5^mol/L) group. (**3A**) Entresto was used for 1 h before Ang II stimulation for 24 h, and then the expression of PCNA and MMP-9 at mRNA level was detected by qRT-PCR. (**3B**) Entresto was used for 1 h before Ang II stimulation for 24 h, and then the expression of PCNA and MMP-9 at the protein level was observed by Western Blot. (**3C**) This is the quantization diagram. Mean ± SEM (*n* = 3). a: *p* < 0.05, compared with the control group. b: *p* < 0.05, compared with Ang II group

The mRNA expression of PCNA and MMP-9 in three groups were 1.00 ± 0.10, 2.73 ± 0.11, 1.40 ± 0.12, 1.00 ± 0.09, 2.56 ± 0.20 and 1.22 ± 0.02 respectively. There were significant differences among the groups (F = 45.91, 33.32, *P* < 0.01). The protein of PCNA and MMP-9 were 0.52 ± 0.06, 1.03 ± 0.03, 0.63 ± 0.06, 0.55 ± 0.03, 1.11 ± 0.01, 0.58 ± 0.04 respectively. There were significant differences among the groups (F = 52.61, 177.2, *P* < 0.01). Compared with the control group, the mRNA expression of PCNA and MMP-9 in Ang II group were increased (t = 7.75, 7.07, *P* < 0.05), and the protein of them were also increased (t = 10.76, 22.93, *P* < 0.01). Compared with Ang II group, the mRNA expression of PCNA and MMP-9 in Ang II + Entresto group decreased (t = 7.03, 5.78, *P* < 0.05), and the protein expression of them also decreased (t = 8.32, 15.82, *P* < 0.05) (Refer to Fig. 4).

### The gene and protein expression of notch1 and jagged1 in VSMCs

**Fig. 5:**
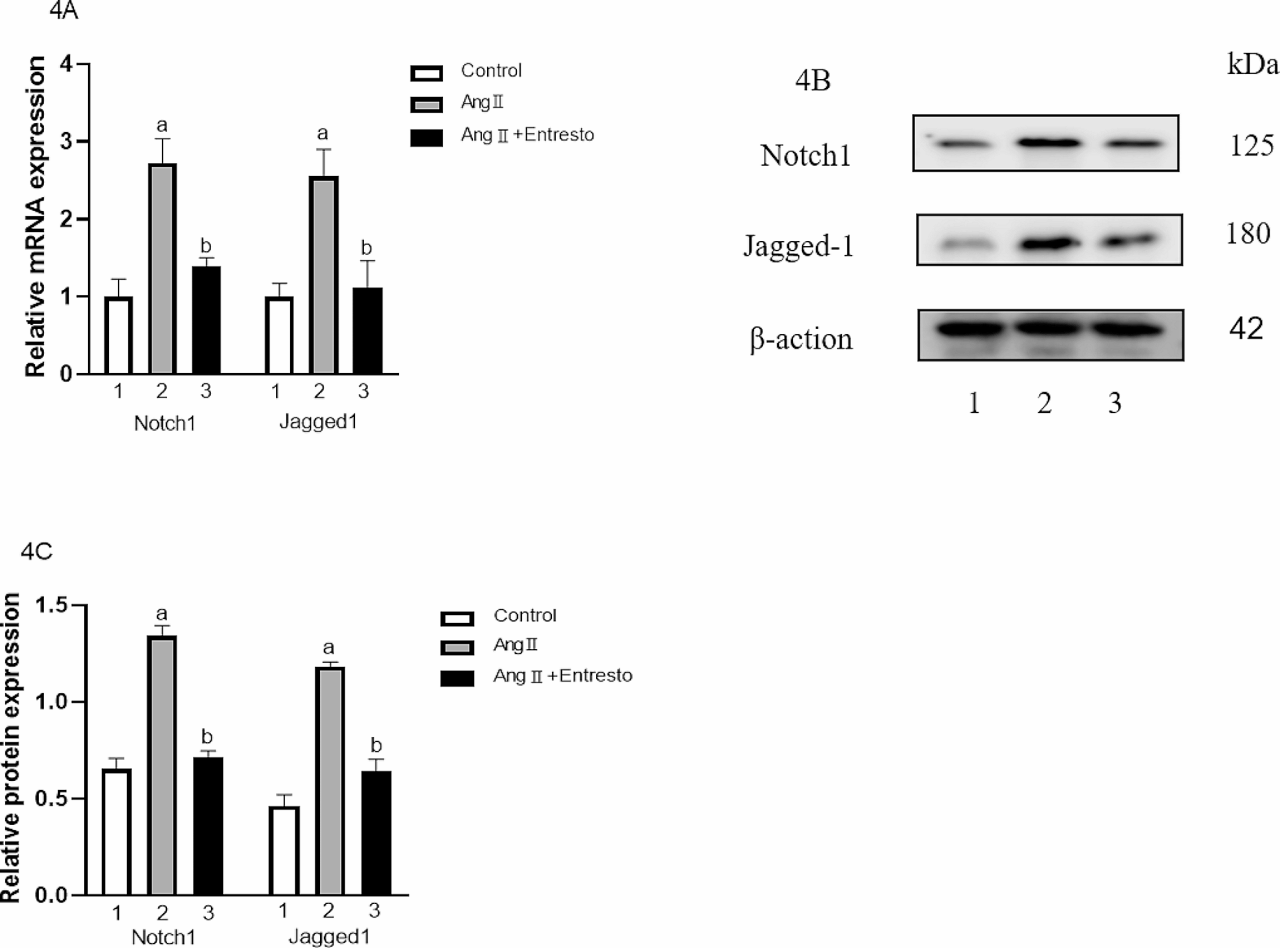
The expression of Notch1 and Jagged-1 in VSMCs. 1: Control group, 2: AngII (10^− 6^mol/L) group, 3: AngII (10^− 6^mol/L) + Entresto (10^− 5^mol/L) group. (**4A**) Entresto was used for 1 h before Ang II stimulation for 24 h, and then the expression of Notch1 and Jagged-1 at mRNA level was detected by qRT-PCR. (**4B**) Entresto was used for 1 h before Ang II stimulation for 24 h, and then the expression of Notch1 and Jagged-1 at the protein level was observed by Western Blot. (**4C**) This is the quantization diagram, mean ± SEM (*n* = 3), a: *p* < 0.05, compared with the control group, b: *p* < 0.05, compared with Ang II group

The mRNA expression of Notch1 and Jagged1 in three groups were 1.00 ± 0.13, 2.73 ± 0.17, 1.39 ± 0.06, 1.00 ± 0.19, 2.66 ± 0.24, 1.42 ± 0.16 respectively. There were significant differences among the groups (F = 45.90, 25.79, *P* < 0.05). The protein expression of Notch1 and Jagged1 were 0.65 ± 0.03, 1.34 ± 0.03, 0.71 ± 0.01, 0.46 ± 0.03, 1.18 ± 0.01 and 0.64 ± 0.03 respectively. There were significant differences among the groups (F = 190.10, 168.30, *P* < 0.01). Compared with the control group, the mRNA expression of Notch1 and Jagged1 in Ang II group increased (t = 7.75, 7.06, *P* < 0.05), and the protein of them also increased (t = 15.67,19.89, *P* < 0.01). Compared with Ang II group, the mRNA expression of Notch1 and Jagged1 in Ang II + Entresto group decreased (t = 7.03,5.12, *P* < 0.05), and the protein of them also decreased (t = 17.41, 14.60, *P* < 0.05) (Refer to Fig. 5).

### The protein expression of p-ERK1/2 in VSMCs

**Fig. 6:**
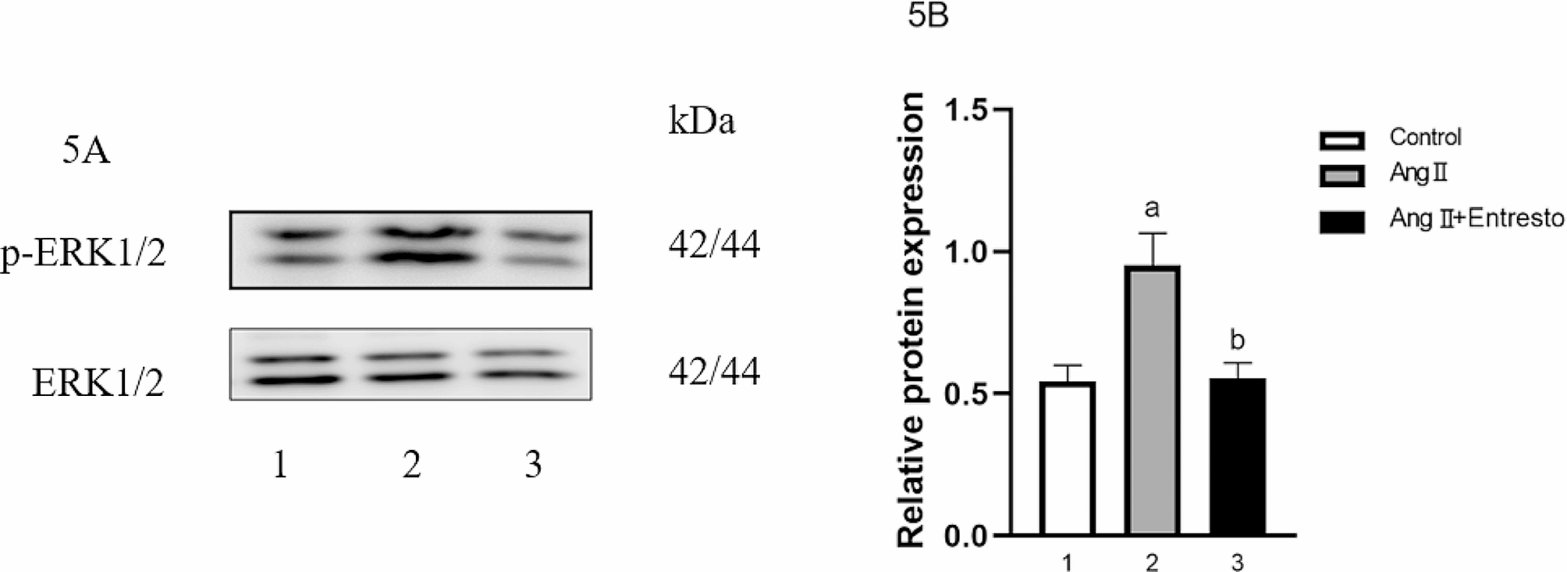
The expression of p-ERK1/2 in VSMCs.. 1: Control group, 2: AngII (10^− 6^mol/L) group, 3: AngII (10^− 6^mol/L) + Entresto (10^− 5^mol/L) group. (**5A**) The expression of p-ERK1/2 at the protein level was detected by Western Blot after Entresto acted for 1 h before Ang II stimulation for 24 h. (**5B**) This is the quantization diagram, mean ± SEM (*n* = 3), a: *p* < 0.05, compared with the control group, b: *p* < 0.05, compared with Ang II group

The protein expression of p-ERK1/2 in three groups were 0.54 ± 0.03, 0.95 ± 0.06 and 0.55 ± 0.03 respectively. There was significant difference among the groups (F = 26.38, *P* < 0.01). Compared with the control group, the protein of p-ERK1/2 in Ang II group was increased (t = 5.66, *P* < 0.01). Compared with Ang II group, the protein of p-ERK1/2 in Ang II + Entresto group was decreased (t = 5.55, *P* < 0.05) (Refer to Fig. 6).

## Discussion

Cardiovascular disease (CVD) is a global public health burden with high morbidity and mortality. CAD has spread all over the world, accounting for 51.7% of the global disease mortality rate, far exceeding the mortality rate of respiratory diseases, cancer, diabetes and other diseases, and has become the main cause of death in the world [19]. PCI is one of the main methods for the treatment of coronary heart disease [20], but ISR has gradually attracted clinical attention.

With the development of materials, there have been bare metal stents (BMS), drug-coated stents (DES) and biodegradable stents (BDS). The main limitation of BMS in clinical treatment is the ISR caused by neointimal hyperplasia, but the subsequent introduction of DES can maximize the improvement of PCI efficacy. DES inhibits neointimal hyperplasia by releasing anti-proliferative drugs, so that the restenosis rate is halved. It is the most widely used stent type [21]. BDS theoretically reduces the influence of the stent itself on the vascular intima on the basis of DES, but the applicable materials of it are still in the exploratory stage [22]. Paclitaxel, sirolimus, and rapamycin are widely used drugs on the surface of stents to counteract restenosis. Although these drugs inhibits neointimal hyperplasia [23], eluting drugs can also slow down the re-endothelialization, delay arterial healing, and may increase the risk of late restenosis. Therefore, it is very important to explore the pathogenesis and prevention strategies of PCI.

The abnormal proliferation and migration of VSMCs is the main pathophysiological mechanism of ISR. Under normal conditions, VSMCs exist in the vascular media to form vascular wall structure and regulate vascular tension to maintain blood pressure [24]. Vascular endothelial injury after stent implantation, subintimal components such as collagen fibers, fibronectin and other exposure, so that platelet activation, aggregation and adhesion in the injury to form local thrombosis [25].Monocytes release inflammatory mediators and chemokines, promote VSMCs from a contractile phenotype into a synthetic phenotype into the intima, increase cell proliferation and migration [26]. VSMCs secrete a large number of platelet-derived growth factor (PDGF), α-fibroblast growth factor, transforming growth factor-β and other cytokines and extracellular matrix [27], pathogenic neointimal hyperplasia process accelerated, leading to the occurrence of ISR. The proliferation and migration of VSMCs were significantly increased after Ang II stimulation.

Notch signaling pathway is mainly composed of ligands, receptors and intracellular effector molecules. In VSMCs, Notch1, Notch2, Notch3 and Jagged1 are dominant [28], while Notch4 is mainly expressed in endothelial cells and endocardium and it has not been reported in VSMCs [29]. Multiple Notch signaling are expressed throughout the cell cycle of VSMCs. In the vascular, endothelial cells express Jagged-1 after receiving the initial signal from the outside world, which binds to the receptor of adjacent VSMCs and activates the Notch signal in a feedforward manner. This interaction enables the Notch signal to propagate in the VSMCs layer through a process called transverse sensing and promotes the differentiation of VSMCs [30].

Yuxin Li et al. found that Notch1 can mediate VSMCs proliferation and neointimal formation after vascular injury by constructing Notch1 heterozygous deficient mice, which may be beneficial to the prevention of vascular proliferative diseases [31]. Anna Chiarini et al. found that Jagged1/Notch1 promoted the adhesion, differentiation and migration of VSMCs through the study of sporadic non-syndromic thoracic aortic aneurysms [30]. Therefore, Notch signaling pathway plays an important regulatory role in the proliferation and migration of VSMCs [32]. Our experimental results showed that the expression of Notch1 and Jagged1 increased after Ang II stimulated VSMCs proliferation, indicating that Notch1/Jagged-1 was involved in the proliferation of VSMCs.

ERK1/2 is the most important pathway mediating cell proliferation effects, and is involved in signal transduction from the plasma membrane to the nucleus [10]. After activation of ERK1/2 into p-ERK1/2, it enters the nucleus and acts on transcription factors such as Elk-1, c-myc, c-fos, c-jun, NF-κB [33]. Nuclear transcription factors and other protein kinases are phosphorylated, thereby regulating the transcription of related genes, causing changes in the expression or activity of specific proteins, and ultimately affecting cells to produce specific biological effects [34].

Studies have shown that AngII-induced VSMCs proliferation and migration can be mediated by MAPK/ERK pathway. Liraglutide can attenuate high glucose-induced abnormal cell migration, proliferation and apoptosis of VSMCs by activating GLP-1 receptor and inhibiting ERK1/2 and PI3K/Akt signaling pathways [35]. In the VSMCs integrin-α9 deficient mouse model [36], the knockdown of integrin-α9 significantly reduced the activation of ERK1/2 and inhibited the phenotypic transformation, proliferation, migration and other cell activities of VSMCs. Therefore, ERK1/2 is one of the important signaling pathways activated by phosphorylation of various stimulators to produce proliferative effects. Our results showed that the expression of p-ERK1/2 was significantly increased after Ang II stimulation, indicating that the proliferation of VSMCs requires the participation of ERK1/2 pathway.

Valsartan/sacubitril is mainly composed of Sacubitril and Valsartan in a 1:1 ratio. The two are not simply combined, but exist in the form of sodium salt complex. This combination can solve two pathophysiological mechanisms: RAS activation and decreased sensitivity to NPs [37]. Valsartan/sacubitril was initially developed for the treatment of heart failure. Subsequent studies have found that Valsartan/sacubitril also has therapeutic effects on diseases such as hypertension, ventricular arrhythmia and pulmonary hypertension. However, the therapeutic effect of Entresto on ISR has not been reported. In this study, CCK8 and the scratch wound assay showed that valsartan/sacubitril affected the proliferation and migration activity of VSMCs induced by Ang II, and decreased the expression of proliferation index PCNA and migration index MMP-9. PCNA is a commonly used index to evaluate the state of cell proliferation, which can be expressed in all cell cycles [38]. MMP-9 belongs to the matrix metalloproteinase family, which can degrade the vascular basement membrane and extracellular matrix [39], and is an important marker in the process of cell migration. The study found that overexpression of MMP-9 can promote the migration of VSMCs to the intima and reduce the content of intima matrix [40]. Therefore, the results of this experiment indicate that valsartan/sacubitril can inhibit the proliferation and migration of VSMCs induced by AngII.

In addition, this study used Entresto to treat VSMCs before Ang II stimulation to observe whether Entresto could affect Jagged-1/Notch1 signaling and ERK1/2 pathway. The results of this experiment showed that compared with Ang II group, the mRNA and protein of Notch1 and Jagged1 in Ang II + Entresto group were significantly decreased, and the protein of p-ERK1/2 was also decreased, indicating that valsartan/sacubitril could down-regulate the expression of Notch1/Jagged1 signal and ERK1/2 pathway. Because these two signaling pathways are involved in the proliferation and migration of VSMCs, it is suggested that valsartan/sacubitril may inhibit the proliferation and migration of VSMCs through Notch1/Jagged1 and ERK1/2 signaling pathways. However, the interaction in these two signaling pathways remains unclear. According to experimental research, Notch can not only affect the expression of ERK1/2 as an upstream signal, thus affecting the life activities of cells, but also be regulated by ERK1/2 [41].

In addition to the Notch and MAPKs signaling pathway studied in this experiment, NF-κB, phosphatidylcholine-phospholipase C and protein kinase C, the Ca^2+^-calmodulin-Ca^2+^/calmodulin-dependent protein kinase signaling pathway, phosphatidylinositol 3’-kinase signaling pathway and TGF-β1/Smad signaling pathway are involved in the phenotypic transformation, proliferation and migration of VSMCs [42]. Whether these signaling pathways are involved in Entresto’s inhibition of VSMCs proliferation has not been studied experimentally. In general, there are few studies on the effect of valsartan/sacubitril on the migration and proliferation of VSMCs and whether it has potential value in the prevention and treatment of restenosis after PCI. Its specific signal transduction mechanism, in vivo research results and application in clinical practice are still unclear. Our in vitro study found for the first time that valsartan/sacubitril may inhibit the proliferation and migration of VSMCs through Notch signaling and ERK1/2 pathway, providing new ideas for valsartan/sacubitril to develop drugs for the prevention and treatment of ISR in clinical practice.

## Conclusion

Based on these results, we concluded that sacubitril/valsartan can inhibit the proliferation and migration of AngII-induced VSMCs, and these effects may be related with activation of Notch1/ Jagged-1 and ERK1/2 signaling pathway. This study deepens our understanding of the therapeutic effect of valsartan/sacubitril, and provides new ideas for its clinical development of drugs for the prevention and treatment of ISR. However, there may be errors between the growth environment of HA-VSMCs in vitro and that of HA-VSMCs in vivo, so more animal and clinical trials are needed to verify our results.

### Electronic supplementary material

Below is the link to the electronic supplementary material.

Supplementary Material 1

Supplementary Material 2

## Data Availability

The data used in this study are available from the corresponding author on reasonable request.
