# Supplementary figures and images for: Sacubitril/valsartan inhibits the proliferation of vascular smooth muscle cells through notch signaling and ERK1/2 pathway
Source: BMC Cardiovasc Disord. 2024 Feb 14;24:106. doi: 10.1186/s12872-024-03764-8 (PMC10865611; doi:10.1186/s12872-024-03764-8)

Figure 2A

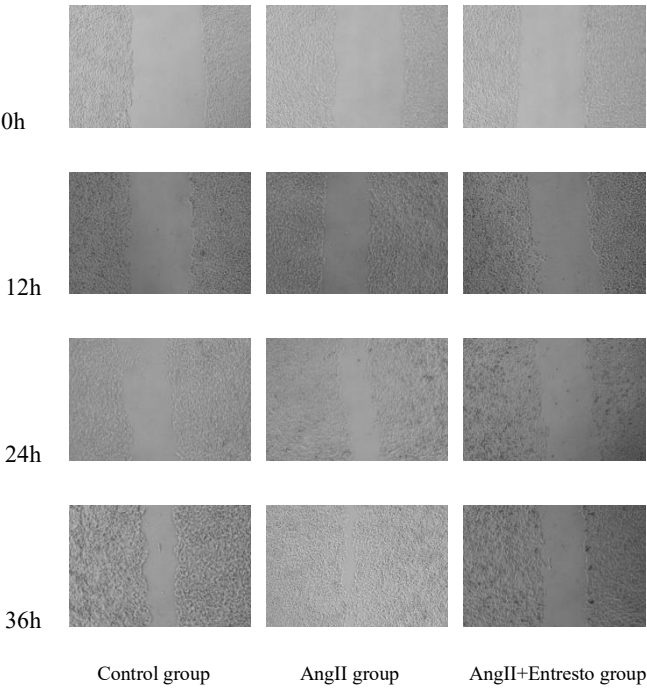

Figure 3B

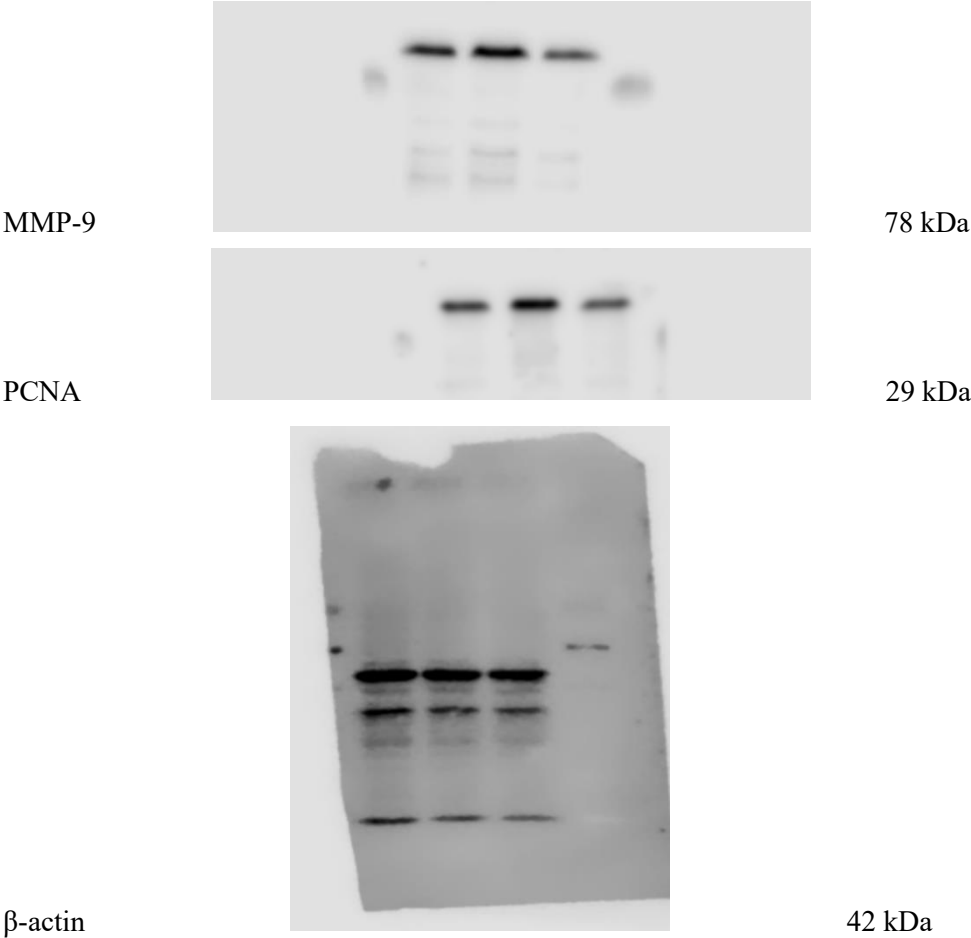

Figure 4B

Notch1

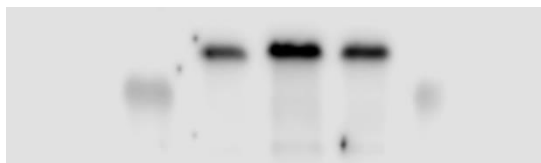

125 kDa

Jagged-1

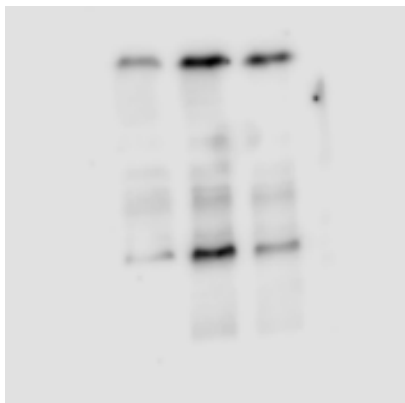

180 kDa

$\beta$ -actin

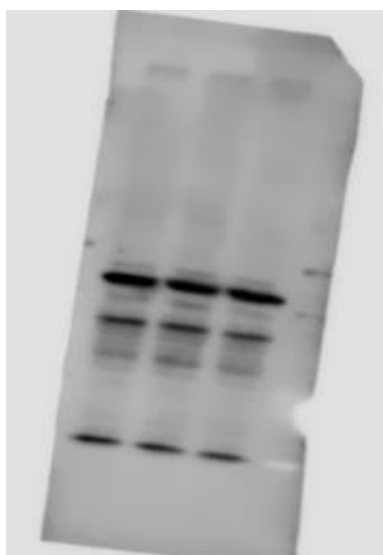

42 kDa

Figure 5A

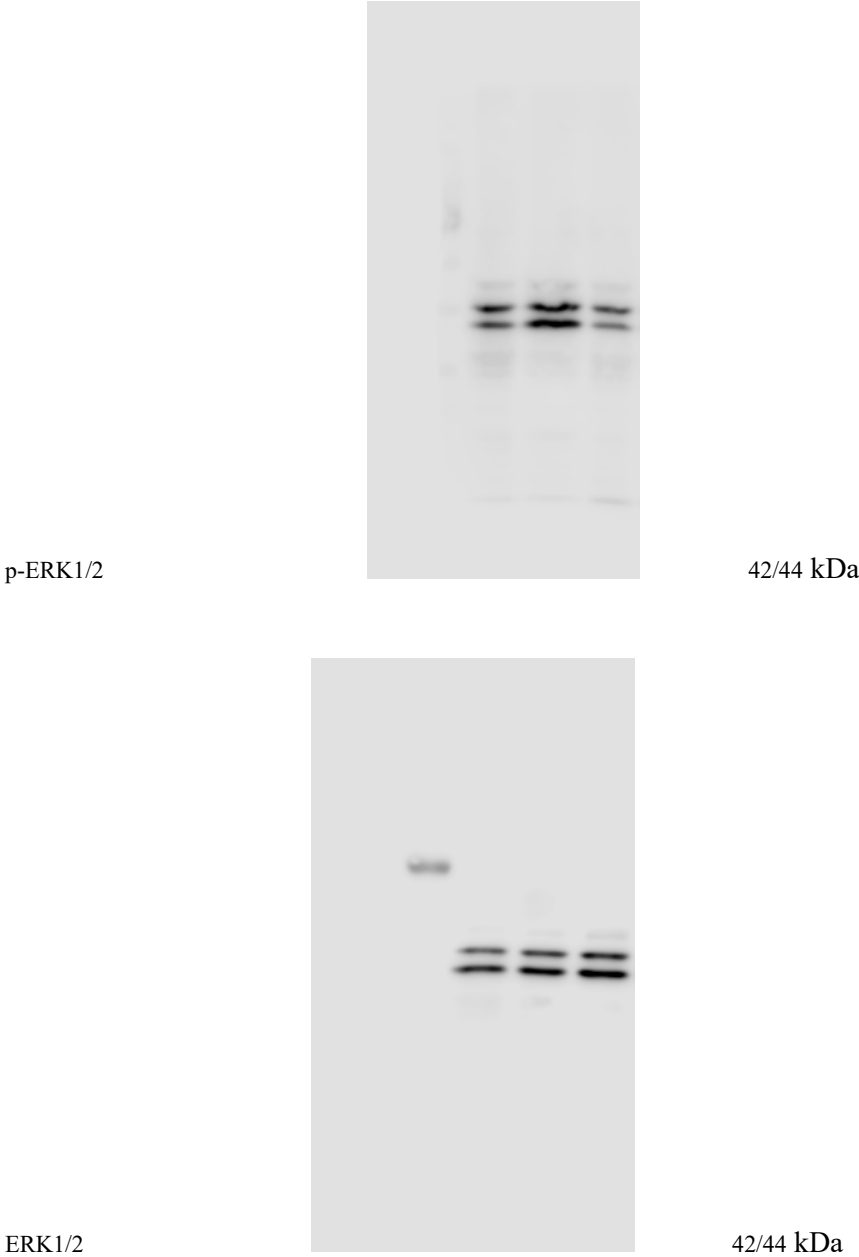

Supplement: Supplementary file 1 — Supplementary Material 1 [file 12872_2024_3764_MOESM1_ESM.pdf]
