# Supplementary material for: Sacubitril/valsartan inhibits the proliferation of vascular smooth muscle cells through notch signaling and ERK1/2 pathway
Source: BMC Cardiovasc Disord. 2024 Feb 14;24:106. doi: 10.1186/s12872-024-03764-8 (PMC10865611; doi:10.1186/s12872-024-03764-8)
Supplement: Supplementary file 2 — Supplementary Material 2 [file 12872_2024_3764_MOESM2_ESM.docx]

The protocol for cutting images is as follows:

As previously stated, we have provided the original image of all blots. Prior to enhanced chemiluminescence visualization of protein bands, the membrane is carefully trimmed to the designated area around the target protein according to its Marker. While the edge of the membrane is clearly visible in the collected images, it exhibits a similar color to the background, resulting in a challenging distinction between the membrane edge and the background. This difficulty in distinguishing the membrane edge from its background primarily accounts for the appearance of closely cropped blots. Unfortunately, due to limited data availability and technological constraints, this issue cannot be rectified at present. Please be advised that each blot was carried out in accordance with the standardized protocol followed in our laboratory.
